# Parental germline mosaicism in genome-wide phased *de novo* variants: Recurrence risk assessment and implications for precision genetic counselling

**DOI:** 10.1371/journal.pgen.1011651

**Published:** 2025-03-31

**Authors:** François Lecoquierre, Nathalie Drouot, Sophie Coutant, Olivier Quenez, Steeve Fourneaux, Fanny Jumeau, Nathalie Rives, Françoise Charbonnier, Céline Derambure, Anne Boland, Robert Olaso, Vincent Meyer, Jean-François Deleuze, Alice Goldenberg, Anne-Marie Guerrot, Camille Charbonnier, Gaël Nicolas

**Affiliations:** 1 Univ Rouen Normandie, Inserm U1245 and CHU Rouen, Department of Genetics and Reference Center for Developmental Disorders, F-76000, Rouen, France; 2 Univ Rouen Normandie, Inserm, U1239 NorDIC, Team Adrenal and Gonadal Pathophysiology, Rouen University Hospital, Reproductive Biology Laboratory-CECOS, F-76000, Rouen, France; 3 Université Paris-Saclay, CEA, Centre National de Recherche en Génomique Humaine (CNRGH), 91057, Evry, France; Baylor College of Medicine, UNITED STATES OF AMERICA

## Abstract

De novo mutations (DNMs) have a significant impact on human health, notably through their contribution to developmental disorders. DNMs occur in both paternal and maternal germlines via diverse mechanisms, including parental early embryonic mosaicism, at high recurrence risk for subsequent pregnancies through germline mosaicism. This phenomenon has been studied mostly on isolated pathogenic variants, but its contribution to genome-wide phased variants in individual genomes is underexplored. We aimed to categorize DNMs and their recurrence risk by detecting and phasing a large set of DNMs via short- and long-read genome sequencing followed by systematic deep sequencing of parental blood and sperm DNA. We detected an average of 85.6 DNM per trio (n=5 trios), with an expected paternal bias of 80%. Targeted resequencing of parental blood and sperm (depth>5000x) revealed 20/334 parental germline mosaics (2–5 per trio) with variant allele fractions (VAFs) ranging from 0.24% to 14.7%, including 7 that were detected in paternal sperm exclusively (1–2 per trio). Owing to paternal bias, maternally phased variants were 3.4x more likely to be mosaic in blood. VAF in sperm samples was used as an indicator for the risk of recurrence of paternally phased DNM. Fourteen variants (out of 244, 5.7%) exhibited detectable sperm mosaicism, while the remaining 230 showed no evidence of mosaicism. Sperm sequencing therefore enabled a precise quantification of the recurrence risk of most individual DNMs. We predict that the use of long-read genome sequencing in genomic medicine will enable the critical step of variant phasing, improving the genetic counselling of rare diseases mediated by DNMs.

## Author summary

Mutations found in an individual but absent in their parents, called *de novo* mutations, occur in every individual. In some instances, they can significantly impact health, especially in severe pediatric disorders. These mutations typically do not occur in the child but were already present in the initial sperm cell or oocyte. The timing of the mutation affects its distribution within families, influencing genetic counseling. Mutations occurring solely in sperm or egg cells are “one-off events” with no recurrence risk. By contrast, mutations arising earlier in a parent’s germline can lead to germline mosaicism, where some germ cells carry the mutation while others do not. This mosaicism poses a risk of recurrence, as the likelihood of variant transmission is influenced by the proportion of affected gametes—a factor rarely assessed in routine genetic testing. Here, we traced the origins of *de novo* mutations in child-parent trios using combined genomic analyses. We focused on identifying germline mosaicism, where a fraction of the child’s mutation is detectable in parental blood or sperm. Our findings provide new insights into the origins and recurrence risks of these mutations.

## Introduction

*De novo* mutations are defined as variants in an individual that are absent from their parents’ genomes, reflecting the germline’s mutability. While all types of variants may occur *de novo*, sequence variants including single nucleotide variants (SNVs) and small insertions deletions (indels) are both common in our genomes, in the range of 60–80 per individual [1,2] and have high impact on health. Indeed, while *de novo* SNVs and indels (thereafter referred to as *de novo* mutations, DNM) are a natural and evolutionary constrained phenomenon [3], they represent a major source of genetic diseases [4]. It has been estimated that approximately one birth in 300 is subject to a severe developmental disorder caused by a DNM in the coding sequence [5]. Trio-based genome sequencing studies have shown high paternal bias, as, on average, 75–80% of DNMs occur on the paternal haplotype, highlighting significant differences in mutability between female and male germlines [1,2,6]. DNMs also exhibit strong paternal age effects. Paternal age at conception is a major determinant of the number of DNMs, while maternal age also plays a role, though to a lesser extent [2,7]. DNMs represent a composite assembly of distinct types of mutational events regarding the timing and the cells in which they appear along the germline, from the zygote to the germ cells in both sexes [8,9]. The magnitude of paternal bias and the paternal age effect implies that mutational events occurring in spermatogonia during adult men’s spermatogenesis are a common cause of DNMs. After this type of mutational event, 50% of the haploid sperm cells produced by mutated spermatogonia are expected to harbour the variant. However, since sperm are produced from millions of spermatogonia, the probability that the same mutation recurs in multiple children (i.e., originating from the same cell) is considered negligible [8]. In contrast, DNMs can result from events occurring in early embryonic cells in one parent [10]. In these situations, mutations may be present in a significant proportion of germ cells (i.e., quiescent oocytes or spermatogonia) and therefore be at high risk of recurrence for future pregnancies.

These two types of mutational events in adult germline and early embryonic cells exemplify the heterogeneity of DNM events in terms of both the mechanism and the risk of recurrence in siblings. This latter property has major implications for genetic counseling in DNM-mediated genetic diseases [11]. The phenomenon of germline mosaicism has long been recognized and has led to the widely accepted understanding that *de novo* variations carry a recurrence risk of approximately 1% for subsequent pregnancies [12]. Many families in which a child carries a severe genetic disease caused by a DNM worry about possible recurrence in subsequent pregnancies and frequently resort to invasive fetal genotyping procedures [13]. However, this 1% estimate represents an average between a majority of families with negligible or no recurrence risk, notably following spermatogonial events (or more broadly “one-off” events, [9]), and families at high risk of recurrence in the case of germline mosaicism. Given the considerable impact of DNMs in certain pathologies and their increased detectability owing to sequencing advances, a finer stratification of DNMs according to mutational event type is needed for clinical care.

One key step in the biology of germline development is the individualization of the germ line from the soma. This phenomenon, called primordial germ cell specification (PGCs), occurs early during human embryogenesis at approximately embryonic day 17 [14] and leads to the specification of 20–40 cells [15] after approximately 10–15 mitotic divisions. Variation occurring before this stage may spread to both the germline and the soma, in the form of “mixed somatic and germline mosaicism” [16] detectable in somatic tissues, whereas variants occurring after PGCS can be clonal only in the germline (“confined germline mosaicism”). Many studies have aimed to assess the recurrence risk of specific pathogenic variants by detecting these two kinds of germline mosaicism via deep sequencing of somatic or sperm samples (S1 Fig and S1 Table). In contrast, few studies have systematically analysed genome-wide DNMs for parental mosaicism, and the prevalence of low-level confined germline mosaicism is underexplored.

In this study, we aimed to categorize a set of genome-wide DNMs by (i) detecting DNMs and systematically phasing them via long-read genome sequencing, (ii) performing targeted deep sequencing of parental blood samples and (iii) performing targeted deep sequencing of paternal sperm samples. This workflow led to the fine mapping of the origin of *de novo* variations in 5 individuals, and to characterizing the risk of recurrence of paternal *de novo* variations.

## Results

### Establishment of a set of high-quality phased *de novo* mutations

**Fig 1:**
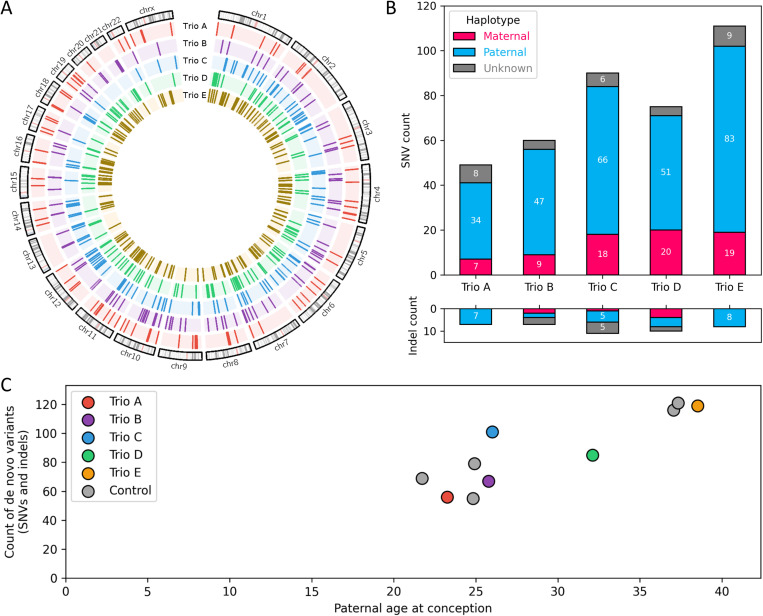
Detection and phasing of *de novo* SNVs and indels recapitulate established DNM properties. DNMs were called on trio short-read genome data sequenced to a 40x target depth, and 90% could be phased onto a parental haplotype using long-read genome sequencing. Count of DNM per individual, proportion of paternal variants (paternal bias), and the observation of expected paternal age effect are used as quality controls for *de novo* variant isolation. A. Genomic distribution of high-quality *de novo* variants. B. Count of *de novo* variants per individual stratified by parental haplotype and variant type. C. Paternal age effect. *De novo* variants detected in 5 additional control trios via similar methods [54] are depicted in grey.

We used short-read genome sequencing to call a set of 428 high-confidence DNMs in five families (S2 Table), ranging from 56 to 119 per individual, with a mean of 77 SNVs and 9 indels (Fig 1). Targeted sequencing of smMIP (single molecule molecular inversion probes) libraries on 349 variations accessible to a MIP design was primarily performed to detect mosaic events, but also served as an independent estimation of precision of *de novo* variant calling. SmMIP sequencing revealed a very low false positive rate, with only 1 variant that appeared to be inherited and 348 true *de novo* variants. However, it is likely that false positive rates would be higher in more complex genomic regions where a design was not possible. By using Nanopore long-read genome sequencing data, we successfully phased 90.5% of the DNM, 80% of which were assigned to the paternal haplotype (ranging from 70 to 85%). By restricting the analysis to short-read sequencing data, only 34% of the variants could be phased, highlighting the expected superiority of long-read sequencing; however, the same paternal bias (79%) was retrieved from short read data only. The paternal age effect was visible for all DNMs (Fig 1C) and for phased DNMs (S2 Fig). Single-base substitution analysis revealed two standard “clock-like” mutational signatures at the expected rates: SBS5 (67%) and SBS1 (24%) (S3 Fig). Three percent of *de novo* variants (13/428) were in mutational clusters (i.e., variants located within a genomic distance of less than 20 kb from each other), and analysis of these clusters also revealed the expected properties, including variant counts, genomic distribution and a biased Ti/Tv ratio (S4 Fig). In summary, we reliably detected *de novo* variants in these genomes, which recapitulated the known properties of *de novo* variants.

### *De novo* variants resulting from parental germinal mosaicism are detectable in every genome

**Fig 2:**
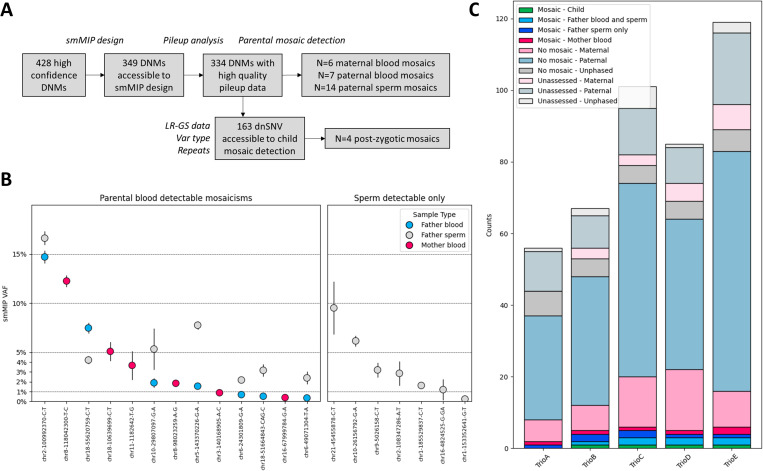
Contribution of parental mosaicism to genome-wide DNMs. *De novo* variants detected in five trios were systematically assessed in parental blood and paternal sperm for mosaicism using high-depth sequencing after single-molecule molecular inversion probes (smMIP)-based enrichment. A. Flowchart for mosaic variant identification. B. Variant allele fraction (VAF) in blood and sperm of confirmed parental mosaicism. For each paternally derived variant, the VAFs for the blood and sperm samples are displayed. The seven variants on the right panel correspond to sperm detectable only with no evidence of blood mosaicism. C. Contribution of mosaics to DNM counts for each trio. Notably, the child (postzygotic) mosaicisms are underestimated since they have been assessed for <50% of all variants (see A).

We used parental blood samples as the source material to detect pre-PGCs embryonic mosaicism and paternal sperm to additionally detect post-PGCs events. Parental mosaicism was assessed in these samples by the measure of variant allele fraction (VAF) using targeted deep sequencing. From the 428 DNMs, 334 were accessible to a smMIP design and had high-quality sequencing pileup data (Fig 2A and S3 Table). The mean smMIP sequencing depth (after deduplication, one x per high-quality read pair) was 5557x, 8314x, and 5755x for child blood, parental blood and paternal sperm samples, respectively. For each position, the four other families served as controls (16 samples in total) to model sequencing noise. Median VAF in controls was 0.022%, indicating limited sequencing noise. Candidate parental mosaicism was called if the VAF differed significantly from the sequencing noise in controls and subsequently confirmed by an independent smMIP experiment. In total, 20/334 variants presented evidence of parental mosaicism (6.0%), including 13 that could be detected in parental blood, and 7 only detected in sperm (Fig 2 and S4 Table). We found that every child carried at least one DNM that was detectable in parental blood (1–4, average 2.6), with VAFs ranging from 0.35% to 14.7%. Parental blood mosaicism indicates early, pre-PGC mutational events occurring before the sexual differentiation of the germline and are therefore likely to be equally common in the paternal and maternal germlines. In line with this, we found similar counts of paternal and maternal mosaics (7 and 6, respectively). However, maternally phased variants were 3.4x more likely to display blood mosaicism than paternally phased variants (6/62=9.7% versus 7/244=2.9%, respectively, Fisher test p=0.0289, S5 Fig), in line with the “dilution” of paternally phased *de novo* variants by events occurring during spermatogenesis [17]. In accordance with the mandatory transmission of mosaic variants to the children in this study, all paternal mosaic variants detected in blood were also detectable in sperm. For these shared mosaic variants, VAFs were often greater in sperm than in blood (6 out of 7 shared mosaicisms). This trend, though not statistically significant (median difference 2.0%, paired Wilcoxon signed-rank exact test, p=0.1094; S6 Fig), is consistent with the findings of previous studies [9,18] and can be attributed to selection bias, as the included variants have all been transmitted to one child.

Germline mosaicism can also occur after PGCs and is therefore only detectable in the germline. Deep sequencing analysis of paternal sperm samples also revealed this type of event in every trio (1–2 events per trio, average 1.4). Consistent with a later occurrence in paternal embryonic development, the point estimate of the VAF of such DNMs identified in sperm only was lower than the sperm VAF of variants also detectable in paternal blood samples (median 2.9% versus 4.2%, respectively), in line with previous observations [19], although the difference was not significant due to the limited sample size (Mann‒Whitney U test p=0.3176). The VAF of mosaicism confined to sperm ranged from 0.24% to 9.5%.

### Detection of postzygotic mutations through a combined approach

Postzygotic variants in a child theoretically pose no risk of recurrence. Therefore, similar to parental embryonic mosaicism, identifying this subtype of DNM is beneficial for accurate genetic counseling. We detected high-confidence postzygotic mosaicism in the probands by using concordant calls from (i) smMIP deep sequencing in children, in which we looked for variants with VAFs deviating from 0.5, and (ii) long-read genome sequencing data. Although both sequencing depth and error rates are in theory suboptimal for detecting mosaicism from long-read data, we exploited the phasing information by focusing on haplotype-specific VAF, corresponding to the VAF of the variant within the mutated haplotype. While this percentage is supposed to be 100% in nonmosaic variants, deviation from 100% indicates a likely mosaicism (S7 Fig). Although this approach was only possible for a subset of variants, we detected 4/163 postzygotic variants (2.45%, Fig 2A and S4 Table), which would translate to an estimated number of 10.5 postzygotic variants in our dataset.

### Recurrence risk assessment of paternally phased variants

Although most of the variants identified in this study are likely neutral, we leverage these data as a proxy to explore the recurrence risk of pathogenic variants, even though certain pathogenic variants may display distinct characteristics regarding mosaicism and resist broader generalization (see Discussion). To assess the recurrence risk in future pregnancies, we hypothesized that the VAF in sperm of paternally phased variants reflects the actual recurrence risk for this subset of variants. This assumption implies that (i) the variant does not affect the likelihood of embryo development and that (ii) the proportion of mutated sperm cells, as indicated by the VAF, remains constant over time. Among the 244 assessed paternally phased variants, we found 14 instances of sperm mosaicism (5.7%), 13 of which had a VAF above the empirical 1% recurrence risk. Sperm VAFs ranged from 0.2% to 16.6%, with a mean value of 4.7% (S4 Table). In contrast, 230 variants did not show evidence for sperm mosaicism, leading to a very low recurrence risk, which is below the mosaic detection rate of our approach. When a null VAF was attributed to variants that did not reach statistical evidence for enrichment over sequencing noise in our analysis, the average VAF and therefore recurrence risk for paternally phased *de novo* variants was 0.27% (95% CI: 0.09–0.46%). Including the raw detected VAF for nonmosaic variants still led to a similar mean VAF of 0.32% (95% CI: 0.13–0.51%), indicating both (i) a low magnitude of sequencing noise and (ii) a limited impact on recurrence risk assessment of true mosaics that we would have failed to distinguish from background noise. Taken together, our results based on sperm mosaic detection revealed a low overall risk of recurrence for paternally phased *de novo* variants, stratified into ~5% of variants at high risk (i.e., greater than 1%) and ~95% of variants at null or very low risk.

## Discussion

### Embryonic mosaicism is a common source of DNMs

To explore the timing of mutational events in the human germline, we used a three-step method to detect genome-wide DNMs, attribute a parental haplotype and assess parental blood and sperm mosaicism in five individuals. We found that parental embryonic mosaicism is a common source of DNMs that are detectable in every genome. Pre-PGC events (detected in blood) contributed to 3.90% of all DNMs analyzed (13/334) and appeared equally distributed on paternal and maternal haplotypes. They accounted for n=2.0 (95% CI: 0.6–3.6) and 1.7 (95% CI: 0.6–3.0) events per child, respectively, after adjustment for detectability. By an innovative method based on “haplotype-specific VAF” on long-read data, we also identified four instances of child post-zygotic mosaicism. It is now well established that a high proportion of post-zygotic *de novo* events may present with a VAF of around 50% [17,20,21], greatly impacting the sensitivity of VAF-based detection approaches. Indeed, detection of post-zygotic variants in 70 individuals using a VAF-independent three-generation pedigree method have shown ~6% of *de novo* variants with a VAF > 0.2 are in fact post-zygotic [20].

Combining parental and child post-zygotic mosaicism led to the low estimate of 5.1% of assessed *de novo* variants (17/334) arising at a pre-PGCs embryonic stage in either generation. This proportion appears remarkably high given the brief embryonic period before PGCs (which occurs around 17 post fertilization [14]), in comparison to the duration of a generation in which DNMs can occur. This observation can be attributed to the pronounced hypermutability of the first few cell divisions after the zygote, which has recently been detected via multiple approaches [22]. This hypermutability coincides with rapid cellular divisions termed “cleavages” without the G1 or G2 phase and the suppression of the cell cycle checkpoint. This special cellular state may be prone to mutations, explaining that this short period of time is critically enriched in DNM.

Altogether, we found that 4.2% of assessed variants (14/334) could be detected as sperm mosaicism. This detection rate exceeds a previous estimate based on 200× whole-genome sequencing [19], where 2.3% of *de novo* variants (21/912) were identified as paternal sperm mosaicism, likely due to the greater sensitivity of our smMIP-based deep sequencing approach. Other studies have investigated parental blood and paternal sperm mosaicism for *de novo* variants, often focusing on pathogenic variants (S1 Fig). While some of these studies have reported higher mosaicism rates, differences could arise from several factors, including assay sensitivity, a potential predisposition of some pathogenic variants to mosaicism, and possible inclusion biases. We found that 2.9% of paternally phased variants (7/244) were present in sperm but not in blood, defining likely post-PGCs events in the fathers. Another approach to quantify this phenomenon is through genomic data from large pedigrees. In a study using WGS in 33 large Utah families, 3.1% of *de novo* variants were classified as post-PGCs events, as they were shared by at least two siblings, but absent from both parents’ blood [20]. However, this proportion is likely underestimated, as the number of offspring per family is finite. Indeed, we found a similar rate of post-PGCs events by assessing only paternal variants, which can only capture half of expected post-PGCs load. Importantly, both methods share similarities, as they each analyze the fraction of mutated gametes—one directly through sperm sequencing and the other indirectly via resulting individuals. While the pedigree-based approach can assess both parental germlines, sperm sequencing provides access to a much larger pool of gametes, offering greater sensitivity.

### Quantifying the risk of recurrence of DNMs

We used sperm VAF as a proxy for recurrence risk, based on the hypothesis that the proportion of mutated cells correlates with the probability of transmission. Indeed, in a recent study, Breuss et al. examined the transmission mechanics of sperm clones and found that the probability of mosaic events being transmitted to blastocysts after in vitro fertilization depended on sperm VAF [18]. By assessing sperm VAF through deep sequencing, we estimated the average risk of recurrence of paternally phased variants to be 0.27% (95% CI: 0.09–0.46%). We compared this estimate with a model based on the observed recurrence rate of variants in an Icelandic population [17] and found that paternally phased variants from our study were predicted to have a higher mean recurrence risk of 0.55% (S1 Text). We expect that our set of *de novo* variants does not capture all variants that are at risk of recurrence. Indeed, in the work by Decode, in addition to detecting *de novo* variants through a trio approach as we did, the authors further detected variants through a haplotype-based method in large families, allowing the detection of variants with high VAFs in parents (high mosaicism) that would be considered as inherited variants by trio-based methods (“near-constitutional” post-zygotic mosaicisms [21]). Indeed, the authors estimated that the trio-based method would miss approximately half of the variants that actually recurred [17].

The risk of recurrence of maternally derived DNMs is difficult to assess via clonal VAF detection because of the inaccessibility of germline cells that harbour post-PGC variants. However, post-PGC events detectable in bulk analysis of germ cells are expected to occur as very early embryonic events (“peri-PGC”, [23]) in primordial germ cells prior to sexual differentiation. Therefore, this shared biology argues that the absolute count and VAF of oocyte mosaicisms should be similar to those of sperm cells. This assumption would mean that the risk of recurrence for maternally derived variants equals RR_pat_ x α, where RR_pat_ is the risk of recurrence for paternally phased variants and α is the ratio of paternal/maternal counts (S2 Text). With this approach and the value of α=4 in our cohort (80% of paternally phased variants and 20% of maternally phased variants), we estimate the maternal recurrence risk to be 1.09% (95% CI: 0.34–1.84%) and the overall risk of recurrence to be 0.44% (95% CI: 0.14–0.74%). This appears to be lower than the commonly accepted risk of recurrence of 1% for DNMs [12]. Once again, the detection method should be considered, and our estimate concerns DNMs detected by stringent trio-based rules.

### Parental mosaicism in short-read genome data

In this study, we applied a sensitive deep sequencing method to detect parental mosaicism. We used these results as a gold standard to compare the performance of parental WGS VAF alone to detect parental blood mosaicism. Considering only variants with at least 1 alternate read in parental WGS, we would have had surprisingly good performances, with 77% recall and 67% precision (S8 Fig). Notably, this would have captured all the variants with a VAF of >1%. Studies on exome sequencing, which typically has higher read depth than WGS, suggested that an alternate read count ≥ 2 was a good indicator of mosaicism rather than sequencing noise [24]. Our results support that the cutoff of ≥ 1 is suitable to call mosaic candidates for 40x WGS data. Given the significance of parental mosaicism in genetic counseling, we recommend confirming pathogenic variants with ≥1 alternative read in parental samples using a more sensitive orthogonal assay. On a technical note, the DeepVariant VCF did not report any alternative reads in cases where mosaicism levels were relatively high (>10%; S2 Table) and clear alternative reads were visible in the alignments. As a workaround for this limitation, SAMtools mpileup was used to enable VAF quantification in mosaic variants (S8 Fig). In summary, our findings indicate that, even in the absence of deep sequencing data from parental samples, actively examining parental WGS alignments for the presence of at least one alternative read can still be highly valuable for assessing recurrence risk.

### Stratification of recurrence risk with long-read genome sequencing and sperm analysis

While deep sequencing of parental blood samples can identify certain variants with a high risk of recurrence, this approach lacks both sensitivity and precision. We showed that post-PGC events in the male germline, as variants detected in sperm only, were as common as pre-PGC events detected in blood, each representing 7/334 (2.1%) of all assessed DNMs in our dataset. Therefore, sequencing sperm samples appeared twice as sensitive as sequencing blood to identify paternal mosaicism. Furthermore, we observed that shared paternal mosaics exhibited differences in VAF on sperm and blood (S6 Fig) with disparities reaching up to a factor of 6.0, suggesting that blood mosaicism may not accurately represent the risk of recurrence. Studies aiming at reconstituting the phylogenies of early cell lineages through various protocols have shown common asymmetry of contribution of the daughter cells to the soma in the first divisions, likely due to stochastic effects [25–28]. It is plausible that similar stochastic effects drive variations in the contribution of specific cells to the germline, resulting in the observed differences in VAFs between somatic cells and sperm. In summary, sperm appears to be an accessible sample type that can be effectively used to estimate the recurrence risk of paternally phased DNMs.

In contrast to our genome-wide analysis of DNMs, previous studies have focused on assessing the recurrence risk of specific pathogenic *de novo* variants using sperm sequencing [9,29–31](S1 Fig). In a remarkable example of 59 *de novo* variants, the authors applied a general framework consisting of (i) phasing the variants via targeted long-read sequencing and (ii) sequencing multiple parental tissues [9]. In our study, long-read genome sequencing was only used to phase the DNMs called from short-read data because of the low performance of the v9 chemistry of Nanopore in small variant calling. However, recent advancements in long-read sequencing technologies have significantly improved this quality. These improvements enable highly accurate and efficient identification of *de novo* variants [32]. Therefore, the transition from short-read to long-read genome sequencing in future years will likely enable much more systematic phasing of DNMs and therefore benefit the genetic counselling of DNM-associated diseases. With long-read based DNM identification, the pipeline for recurrence risk assessment could be restricted to deep sequencing analysis of paternal sperm for paternal variants. Such a viable approach would lead to a precise estimation of recurrence risk for 80% of DNMs and avoid unnecessary invasive prenatal testing procedures in most of these cases. There is little variation in the VAF of sperm mosaicism over time [19,33], which could corroborate this approach of using VAF as a proxy for the risk of recurrence of paternally phased variants. While techniques of prenatal diagnosis improve and noninvasive techniques (NIPT) become accessible for *de novo* variants [34], the anticipation of the recurrence risk by sperm analysis before any pregnancy could better suit some families and present the advantage of being performed only once versus one NIPT at each pregnancy.

On the other hand, maternally phased *de novo* variants pose challenges for recurrence risk stratification. *De novo* variants on the maternal haplotype have a higher probability of germline mosaicism (S5 Fig), which translates into a higher risk of recurrence. The presence of a low-rate mosaicism in blood in case of a pre-PGCs variant may lead to false positives in NIPT, and gametes are not accessible to precisely detect germinal mosaicism. For these reasons, maternally phased *de novo* variants should more readily prompt invasive foetal sampling when assessing the risk of recurrence of a pathogenic *de novo* variant for a new pregnancy.

### Factors impacting recurrence risk

Parental age at conception is a major determinant of the total count of *de novo* variants by its impact on the mutation load of the adult germline (namely spermatogonia and primary oocytes). Since these DNMs are not at risk of recurrence [8], an advanced age at conception is paradoxically associated with a lower risk of recurrence for a given DNM. This effect was not significant in our study but has been observed with larger sample sizes [17]. Besides parental age and parental haplotype, additional factors should be considered when assessing the recurrence risk of DNMs. Some pathogenic variants in specific genes can lead to a developmental advantage of the wild type or mutant cell over the other [22], leading to biased recurrence risk. For example, selfish mutations affecting the RAS/MAPK pathway occur almost systematically in the paternal adult germline, and even though these mutations lead to spermatogonial clonality, the overall proportion of mutated cells is very limited [35]. In line with this, epidemiological observations have revealed a low risk of recurrence for selfish mutations, questioning the necessity of prenatal diagnostic testing in subsequent pregnancies after the birth of an affected child [36]. In contrast, pathogenic variants in other genes, such as *SCN1A*, appear to be enriched in parental mosaicism and *de novo* recurrence risk [37–45]. Another genomic feature that could be used for recurrence risk assessment might be the presence of the variant in a mutational cluster (i.e., multiple variants within a small genomic interval, typically 20 kb). Many mutation clusters are thought to be derived from age-related changes in the biology of the germline, notably in oocytes [46]. Therefore, clustered variants could be indicative of low recurrence risk. Interestingly, none of the 11 clustered variants in which deep sequencing was performed presented evidence of parental mosaicism. Larger studies are needed to assess the correlation between the risk of recurrence and occurrence in mutation clusters.

## Conclusion

In summary, we present the proportion of genome-wide DNMs mediated by the mechanism of parental embryonic mosaicism. We estimate the average recurrence risk of DNMs detected in WGS trio analysis to be less than 1%. For 80% of the variants mapped to the paternal haplotype, sequencing of paternal sperm samples enabled a more precise assessment of recurrence risk, with 95% of these variants classified as having negligible risk and 5% with a risk greater than 1%.

## Methods

### Ethics statement

This study was approved by the Comité de Protection des Personnes Ouest V (CPP) ethics committee, reference 20/043-2. Informed written consent was obtained from all participants or their legal guardians. The GERMETHEQUE biobank (BB-0033–00081), site of Rouen, provided 5 samples of spermatozoa and their associated data for this project. GERMETHEQUE obtained consent from each patient to use their sperm samples (CPP 2.15.27). The GERMETHEQUE steering committee approved the study design on 17/11/2020. The Biobank has the declaration DC-2021–4820 and the authorization AC-2019–3487. The number of requests made to Germethèque is 20201117.

### Patients and samples

Five trios consisting of one child and both parents were included. The probands were affected by undiagnosed neurodevelopmental disorders (NDD), and the sequencing techniques deployed in this protocol were used to help identify the cause of the disease as a secondary objective, previously reported [47]. Of note, while ref [47] mentions the identification of three diagnosis out of five probands, the recent discovery of *RNU4–2* as a common NDD associated gene [48] allowed a fourth diagnosis on proband D, who carries the most recurrent *RNU4–2* insertion n.64_65insT, which occurred *de novo*. Maternal age at conception ranged from 24.2 to 30.9 years, and paternal age ranged from 24.0 to 39.3 (S2 Fig).

EDTA blood samples were collected from each individual, as well as sperm samples from the five fathers. Paternal age at sperm collection ranged from 34.5 to 45.7 years and translated into 3.4 to 14.3 years after child conception. DNA was extracted from blood via standard procedures for short-read-based sequencing techniques. Longer fragments were also extracted from peripheral blood mononuclear cells (PBMCs) using Revolugen kit for 4 trios and from frozen blood using Circulomics kit for one. Sperm samples were collected into a sterile container (Clinisperm, CML, Nemours, France) directly at the Rouen University Hospital Reproductive Biology Laboratory CECOS after sexual abstinence for 3–5 days according to WHO quality guidelines. A liquefaction time of 20–30 min was allowed before the sperm were frozen in straws (Spermfreeze, dilution ½: one volume of solution for one volume of semen), JCD International Laboratory, Lyon, France). Gradient centrifugation was performed to isolate motile sperm cells from other cell types and cellular debris. A one-layer gradient was prepared using 90% fractions of Puresperm (JCD International Laboratory, Lyon, France) diluted in IVF medium (Origio, CooperSurgical, Måløv, Denmark) and centrifuged at 150 × g for 20 min. Then, the 90% fraction was washed with IVF medium by centrifugation at 350 × g for 10 min. DNA was extracted from the sperm pellet via the TCEP-based method of Wu et al. [49].

### Genome sequencing

Short-read genome sequencing was performed at the *Centre National de Recherche en Génomique Humaine* (CNRGH, Institut de Biologie François Jacob, CEA, Evry, France), using paired-end 150 bp reads on NovaSeq 6000 and targeting an average sequencing depth of ~40x. Actual depth ranged 33-58x across all individual, reaching>40x in all five probands (S4 Table). Long-read genome sequencing was performed by CNRGH on an Oxford Nanopore Promethion system with R9 chemistry after preparation via SQK-LSK109 or SQK-LSK110 ligation kits. Median depth of sequencing across samples was 41x and read length N50 was 13.8 kb (i.e., 50% of sequenced bases belonged to reads>= 13.8 kb). Further details on the short- and long-read sequencing procedures and quality metrics for these five trios are available in ref [47].

### *De novo* variant identification and phasing

*De novo* single nucleotide variant (SNV) and short insertion/deletion (indel) candidates were identified from 43x short-read genome data. Reads were aligned on GRCh38 via BWA, and short variants were called via DeepVariant V1.5 via default parameters for Illumina WGS. A 15 samples multi-vcf was produced using Glnexus. A two-step workflow was applied to isolate high-quality *de novo* variants. First, *de novo* SNV and indel candidates were detected via simple filtration steps using a BCFtools-based custom python script. These filters included genotype (GT=alt in child and ref in both parents), depth (DP > 20 in all three individuals), DeepVariant genotype quality (GQ > 29 in all three individuals), variant allele fraction (VAF > 0.25 in child), exclusion of multiple allelic loci (AD1 + AD2 > 0.7 × DP), and a shift in VAF between each parent and the child of at least 4x. This last requirement was used to avoid the use of strict alt read counts or VAFs in parents and allows for the detection of cases of parental mosaicism. The second step consisted of a manual review of DNM candidate calls via an IGV-based classifier interface. The scripts used for DNM isolation and reviewing are available at SCR_026181. Substitution-based signatures identified by Degasperi et al. [50] were extracted via Signal software [51]. Variant phasing (i.e., identification of the parental haplotype on which the variant occurred) was achieved via short- and long-read data. The long-read genomes were aligned on GRCh38 via Minimap2. SNVs and indels detected from short-read WGS were phased in trios using long-read information by WhatsHap phase. Because the WhatsHap version used did not allow for phasing of *de novo* variants directly, we used a manual method based on manual inspection of long-read haplotypes. Phased VCF was used to add the phase to individual Nanopore reads via WhatsHap haplotag, and a manual review of the alignments was applied for a definite parental haplotype attribution for each *de novo* variant. Variants were also phased using short reads only via Unphazed software [52].

### Targeted deep sequencing

Deep sequencing at DNM positions was performed on child and parental blood samples as well as paternal sperm samples via Single-Molecule Molecular Inversion Probe (smMIP)-based sequencing, similar to previously described methods [53]. SmMIPs are oligonucleotides which contain two target-specific arms that hybridize to flanking regions of the DNA, enabling gap filling, circularization, and subsequent amplification for high-depth target capture. One smMIP was designed around each DNM position via MIPGEN with arms_length_sums = 38 (corresponding to the sum of the length of both target specific arms, in nucleotides) and a varying capture_size from 90-110 (corresponding to the size in nucleotide of both arms + target region). Ten nucleotides of unique molecular identifiers were used (2 × 5 nt) to allow a maximum of 4^10^ (1048576) combinations. Counts of occurrences of extension and ligation probes in the reference genome provided by MIPGEN were used to exclude smMIPs if either one of the two arms had a sequence occurring > 20 times or if both arms had multiple occurrences, and *in silico* PCR (UCSC, default parameters) led to more than one result. The final design of 346 oligos (S5 Table) was produced by IDT DNA technologies. Individual smMIPS were pooled and phosphorylated. An amount of 300 ng of input DNA was used for smMIP capture at a 1:4000 ratio (1 genome copy for 4000 smMIP molecules). The capture product was then amplified and indexed via 16-cycle PCR. Libraries were pooled and sequenced on three high 2x75 flow cells on an Illumina NextSeq 500 sequencer. Deep sequencing reads were aligned to the reference genome, and duplicates were removed via UMI tools. The variant allele fraction (VAF) and sequencing depth were assessed for all the variants in all the samples via SAMtools mpileup launched via a python script.

### Mosaic variant identification and statistics

For each variant, the VAF and sequencing depth were established from the father’s blood, sperm, mother’s blood and controls from the sequencing pool. VAF was defined as the proportion of alt_read_count/(ref_read_count + alt_read_count), which we referred to as ‘Two-allele VAF.’ This measure does not account for other genotypes than ref and alt that may appear in deep sequencing pileups. The controls, used to discriminate mosaicism events from sequencing noise, consisted of a child and three parental samples for the other 4 trios (16 samples total). To detect candidate mosaic variants, VAFs in the father’s blood, sperm or mother’s blood were compared to the VAF in merged controls. To account for extremely low allelic ratios among controls, we adopted a one-sided Poisson test. Owing to phasing, not all three mosaicisms had to be tested at every position. When the child’s variant could be phased to the paternal haplotype, potential mosaicism was searched within the father’s blood and sperm only. When the variant was of maternal origin, potential mosaicism was searched within maternal blood only. When phasing was not possible, the father’s blood and sperm, as well as the mother’s blood, were investigated for parental mosaicism. As a result, a Bonferroni correction was applied to account for a total of 637 haplotype-coherent tests, with the requirement of an overall type-I error threshold of 0.05/637=7.8x10-5 for experiment-wide significance.

Candidate mosaic variants were confirmed on the parental samples via an independent sequencing assay of similar depth using a restricted pool of 40 smMIPs, which were sequenced on a 2x75 flow cell on an Illumina NextSeq 500 sequencer.

## Supporting information

S1 Table Literature review for studies assessing the rates of parental mosaicism. Data displayed in S1 Fig.
(XLSX)

S2 Table De novo variants identified in this study. (XLSX)

S3 Table Raw results of smMIP sequencingThe terms “two allele total” and “two allele VAF” (variant allele frequency) refer to reads supporting either the reference (ref) or alternate (alt) genotype of the variant. Reads containing additional genotypes (third allele or more) are excluded.
(XLSX)

S4 Table Instances of parental embryonic mosaics detected. The terms “two allele total” and “two allele VAF” (variant allele frequency) refer to reads supporting either the reference (ref) or alternate (alt) genotype of the variant. Reads containing additional genotypes (third allele or more) are excluded.
(XLSX)

S5 Table Quality metrics of short and long read genome sequencing. SR: short-read genome sequencing, LR: long-read genome sequencing, SNV: single nucleotide variant, indel: short insertion/deletion.
(XLSX)

S6 Table smMIP design. Ext probe: extension probe. Lig probe: ligation probe. Tm: melting temperature.
(XLSX)

S1 Fig Literature review: studies exploring parental mosaicisms from de novo mutations. This plot displays the proportion of the total count of DNMs which is detected to result from parental mosaicism in blood or paternal sperm. Inclusion criteria were: (i) at least 30 variants investigated, and (ii) a sensitive technique specifically applied to detect parental mosaicism, such as deep NGS or ddPCR. Several studies focused on pathogenic DNMs, including many studies on epileptic syndromes notably caused by DNMs in SCN1A, which often display higher rates of parental mosaicism. Few studies assessed the parental mosaicism rate for genome-wide DNMs with sensitive techniques. Of note, the genome-wide study conducted on paternal sperm cells [19] used 200x WGS, which did not allow the detection of low VAF mosaicism explaining the relatively low proportion of sperm mosaicism.
(PDF)

S2 Fig Parental age effect on phased variants. Phased SNV + indel counts are plotted against parental age at conception. Linear regressions show stronger parental age effect than usually reported, likely due to small sample size.
(PDF)

S3 Fig Single base substitution signatures extraction from the 385 de novo substitutions detected in this study. Signatures were extracted using the Signal interface (https://signal.mutationalsignatures.com/analyse2) based on signatures from Degasperi et al [50]. A. Proportion of trimer substitutions. B. Signatures extracted using the Signal interface. The SBS5 and SBS1 signatures are detected. TSB: transcriptional strand bias. The ‘TSB’ and ‘Deamination’ tags, as well as the ‘Age’ label, are standard annotations of the signatures and do not derive from the inputted DNMs.
(PDF)

S4 Fig Analysis of mutational clusters recapitulate known cluster properties. Clusters were defined as variations separated by a maximum of 20kb and called by https://github.com/francois-lecoquierre/de_novo_tools/blob/main/DNM_cluster_by_sample.py. A. Genomic distribution of clusters. Regions enriched in maternal mutational clusters identified in the literature are shown in pink. Note that the only maternal cluster is present in one of these regions. It is also the largest cluster and contains the most variants (n=3, see C and D). Generated using Tagore software (https://github.com/jordanlab/tagore). B. Types of substitutions of clustered (n=13) versus non-clustered (n=372) variations. The drastic difference in Ti/Tv ratio between clustered and non-clustered variations recapitulates observations on larger trio studies. C. Characteristics of the 6 clusters detected. Note the higher prevalence of paternal clusters, in contrast to literature data in which the number of paternal clusters is equivalent to the number of maternal clusters. This difference is likely due to the small sample size. Of note, the phases of individual SNVs were concordant and have been merged in the “Parent Of Origin” column. D. Representation of the maternal cluster composed of 3 SNVs within the SMARCA2 gene in the hypermutable 9p region. UCSC euro session: https://genome-euro.ucsc.edu/s/francois.leco/RRMUT_maternal_cluster.
(PDF)

S5 Fig Maternally phased variants display higher rate of parental blood mosaicism than paternally phased variants. (PDF)

S6 Fig Shared mosaicisms in fathers: correlation of VAF in sperm versus blood. Two Allele VAF is defined by alt_read_count/(ref_read_count + alt_read_count) and does not integrates reads with other genotypes than ref and alt.
(PDF)

S7 Fig High evidence for four child embryonic mutations. Early embryonic mosaicism was called for a subset of variants using two complementary VAF-related metrics within: (i) high depth smMIP data and (ii) phased long-read genome data. The variant chr2–43736835-C-A is displayed as an example for both these metrics in A) and B). A. smMIP pileup genotyping of the variant chr2–43736835-C-A showing a VAF that deviates from the 50% expected for a homogeneous genotype. B. The same variant as seen in the proband’s long read genome data. The de novo C>A transversion is phased on the maternal haplotype 2, corresponding to the purple reads. Ten maternally derived reads do not harbour the variant, highly suggesting mosaicism. From this example, the ratio of C>A-bearing purple reads over the total count of purple reads defines what we called the haplotype-specific VAF. This metrics is expected to be 100% in samples without post-zygotic mosaicism. C. Detection of child embryonic mosaicisms using the combination of the two VAF-related metrics. From all de novo variants, we extracted a subset of 163 variants with high quality genotypes in child’s sequencing data, both in Nanopore long-read genome data and high depth smMIP sequencing. More specifically, the filters included: (i) SNVs only, (ii) variants with a parental phase determined in long-read data, with a depth of at least 4x on the haplotype bearing the variant, and (iii) variants for which both the extension and ligation arms of the associated smMIP did not lie within a unique repeated element in “RepeatMasker” or “Human Self Chain Alignments” tracks from UCSC, since highly repeated elements were occasionally observed to slightly bias the smMIP-defined VAF by incomplete specificity. The smMIP VAF is centered on 0.5 for heterozygous de novo variants, as expected. The haplotype-specific VAF has been defined as the proportion of alt reads over (ref + alt) reads, only on the mutated haplotype defined by WhatsHap Haplotag. Since both smMIP and Nanopore sequencing exhibit noise in the definition of the VAF, we considered high evidence mosaicisms as the variants with low VAF in both approaches. In this perspective, child mosaics were defined as variants with both a smMIP VAF < 0.45 and a haplotype-specific VAF < 0.9. These thresholds are indicated in pink dotted lines. Four variants meeting these criteria are highlighted. De novo variants with evidence for parental mosaicism are indicated in yellow and serve as negative controls since they are necessarily pre-zygotic.
(PDF)

S8 Fig Correlation between counts of alt reads in parental WGS and parental mosaicism status. Samtools Mpileup was used to quantify the alt read count in parental WGS for the de novo variants included in this study and to compare it to the presence of a mosaicism as detected by deep sequencing. A. Counts of alt reads in parental genomes and mosaicism status. As expected, the presence of ≥ 2 reads in parental genomes appears highly predictive for parental embryonic mosaicism. These counts can be used to establish the performance of at least one alt read as an indicator of parental mosaicism. Recall is defined by the proportion of variants with mosaicism that have ≥ 1 alt read in parental WGS: 10/13 = 76.9%. Precision is defined by the proportion of variant with ≥ 1 alt read that are actually mosaic variants: 10/15 = 66.7%. B. Parental blood mosaicism: true blood VAF against alternate read count in 40x WGS. The 13 mosaicisms confirmed to be present in parental blood are plotted. Alternate read count from parental 40x WGS appears predictive of blood VAF detected by deep sequencing.
(PDF)

S1 Text Prediction of recurrence risk using decode genetics’ de novo mutation recurrence calculator. (PDF)

S2 Text Assessing the risk of recurrence for maternally derived variants. (PDF)
