# Supplementary material for: Parental germline mosaicism in genome-wide phased de novo variants: Recurrence risk assessment and implications for precision genetic counselling
Source: PLoS Genet. 2025 Mar 31;21(3):e1011651. doi: 10.1371/journal.pgen.1011651 (PMC11990764; doi:10.1371/journal.pgen.1011651)
Supplement: S1 Fig — This plot displays the proportion of the total count of DNMs which is detected to result from parental mosaicism in blood or paternal sperm. Inclusion criteria were: (i) at least 30 variants investigated, and (ii) a sensitive technique specifically applied to detect parental mosaicism, such as deep NGS or ddPCR. Several studies focused on pathogenic DNMs, including many studies on epileptic syndromes notably caused by DNMs in SCN1A, which often display higher rates of parental mosaicism. Few studies assessed the parental mosaicism rate for genome-wide DNMs with sensitive techniques. Of note, the genome-wide study conducted on paternal sperm cells [19] used 200x WGS, which did not allow the detection of low VAF mosaicism explaining the relatively low proportion of sperm mosaicism. (PDF) [file pgen.1011651.s007.pdf]

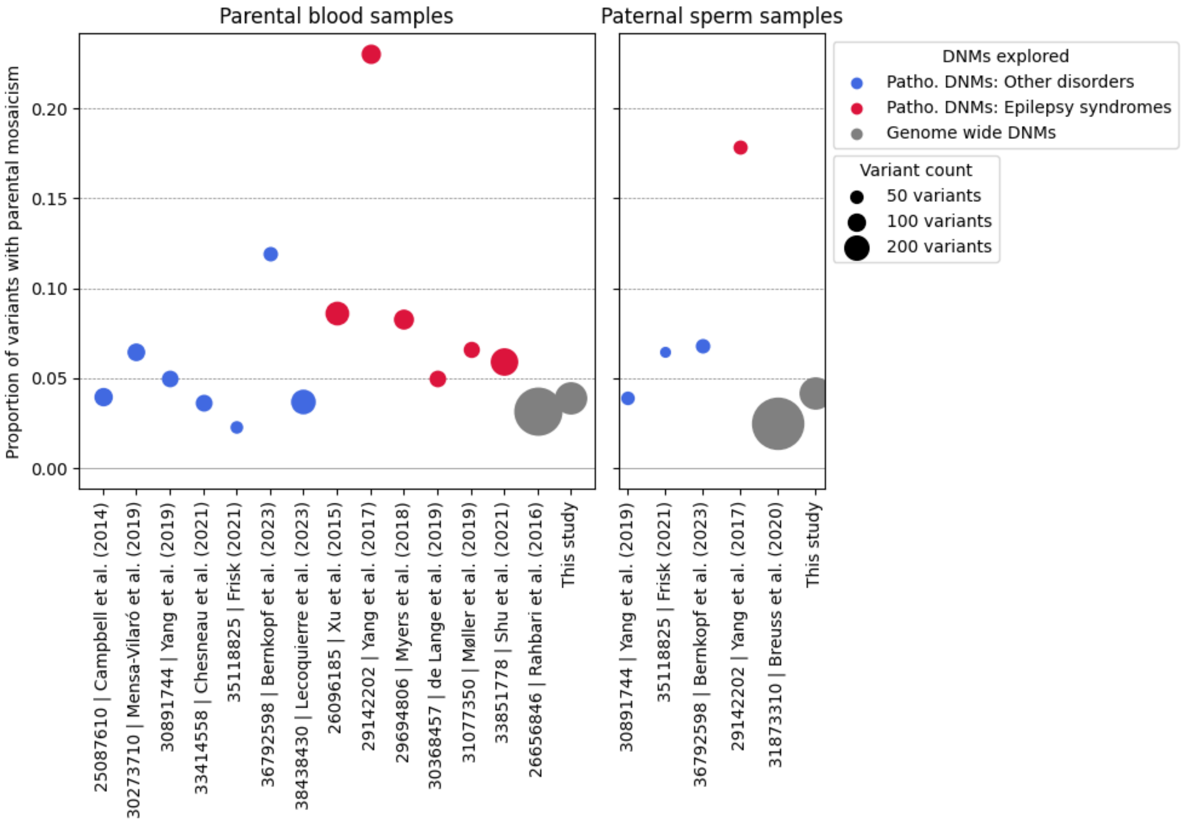

Supplementary Figure 1. Literature review: studies exploring parental mosaicisms from de novo mutations
This plot displays the proportion of the total count of DNMs which is detected to result from parental mosaicism in blood or paternal sperm. Inclusion criteria were: (i) at least 30 variants investigated, and (ii) a sensitive technique specifically applied to detect parental mosaicism, such as deep NGS or ddPCR. Several studies focused on pathogenic DNMs, including many studies on epileptic syndromes notably caused by DNMs in *SCN1A*, which often display higher rates of parental mosaicism. Few studies assessed the parental mosaicism rate for genome-wide DNMs with sensitive techniques. Of note, the genome-wide study conducted on paternal sperm cells [1] used 200x WGS, which did not allow the detection of low VAF mosaicism explaining the relatively low proportion of sperm mosaicism.
References: [1–15] are presented in next page.

1. Breuss MW, Antaki D, George RD, Kleiber M, James KN, Ball LL, et al. Autism risk in offspring can be assessed through quantification of male sperm mosaicism. Nat Med. 2020;26: 143–150. doi:10.1038/s41591-019-0711-0

2. Campbell IM, Yuan B, Robberecht C, Pfundt R, Szafranski P, McEntagart ME, et al. Parental somatic mosaicism is underrecognized and influences recurrence risk of genomic disorders. Am J Hum Genet. 2014;95: 173–182. doi:10.1016/j.ajhg.2014.07.003

3. Mensa-Vilaró A, Bravo García-Morato M, de la Calle-Martin O, Franco-Jarava C, Martínez-Saavedra MT, González-Granado LI, et al. Unexpected relevant role of gene mosaicism in patients with primary immunodeficiency diseases. J Allergy Clin Immunol. 2019;143: 359–368. doi:10.1016/j.jaci.2018.09.009

4. Yang X, Yang X, Chen J, Li S, Zeng Q, Huang AY, et al. ATP1A3 mosaicism in families with alternating hemiplegia of childhood. Clin Genet. 2019;96: 43–52. doi:10.1111/cge.13539

5. Chesneau B, Plancke A, Rolland G, Chassaing N, Coubes C, Brischoux-Boucher E, et al. Parental mosaicism in Marfan and Ehlers-Danlos syndromes and related disorders. Eur J Hum Genet. 2021;29: 771–779. doi:10.1038/s41431-020-00797-3

6. Frisk S, Wachtmeister A, Laurell T, Lindstrand A, Jäntti N, Malmgren H, et al. Detection of germline mosaicism in fathers of children with intellectual disability syndromes caused by de novo variants. Mol Genet Genomic Med. 2022;10: e1880. doi:10.1002/mgg3.1880

7. Bernkopf M, Abdullah UB, Bush SJ, Wood KA, Ghaffari S, Giannoulatou E, et al. Personalized recurrence risk assessment following the birth of a child with a pathogenic de novo mutation. Nat Commun. 2023;14: 853. doi:10.1038/s41467-023-36606-w

8. Lecoquierre F, Cassinari K, Drouot N, May A, Fourneaux S, Charbonnier F, et al. Assessment of parental mosaicism rates in neurodevelopmental disorders caused by apparent de novo pathogenic variants using deep sequencing. Sci Rep. 2024;14: 5289. doi:10.1038/s41598-024-53358-9

9. Xu X, Yang X, Wu Q, Liu A, Yang X, Ye AY, et al. Amplicon Resequencing Identified Parental Mosaicism for Approximately 10% of “de novo” SCN1A Mutations in Children with Dravet Syndrome. Hum Mutat. 2015;36: 861–872. doi:10.1002/humu.22819

10. Yang X, Liu A, Xu X, Yang X, Zeng Q, Ye AY, et al. Genomic mosaicism in paternal sperm and multiple parental tissues in a Dravet syndrome cohort. Sci Rep. 2017;7: 15677. doi:10.1038/s41598-017-15814-7

11. Myers CT, Hollingsworth G, Muir AM, Schneider AL, Thuesmunn Z, Knupp A, et al. Parental Mosaicism in “De Novo” Epileptic Encephalopathies. N Engl J Med. 2018;378: 1646–1648. doi:10.1056/NEJMc1714579

12. de Lange IM, Koudijs MJ, van ’t Slot R, Sonsma ACM, Mulder F, Carbo EC, et al. Assessment of parental mosaicism in SCN1A-related epilepsy by single-molecule molecular inversion probes and next-generation sequencing. J Med Genet. 2019;56: 75–80. doi:10.1136/jmedgenet-2018-105672

13. Møller RS, Liebmann N, Larsen LHG, Stiller M, Hentschel J, Kako N, et al. Parental mosaicism in epilepsies due to alleged de novo variants. Epilepsia. 2019;60: e63–e66. doi:10.1111/epi.15187

14. Shu L, Zhang Q, Tian Q, Yang S, Peng X, Mao X, et al. Parental mosaicism in de novo neurodevelopmental diseases. Am J Med Genet A. 2021;185: 2119–2125. doi:10.1002/ajmg.a.62174

15. Rahbari R, Wuster A, Lindsay SJ, Hardwick RJ, Alexandrov LB, Turki SA, et al. Timing, rates and spectra of human germline mutation. Nat Genet. 2016;48: 126–133. doi:10.1038/ng.3469
