# Supplementary material for: Parental germline mosaicism in genome-wide phased de novo variants: Recurrence risk assessment and implications for precision genetic counselling
Source: PLoS Genet. 2025 Mar 31;21(3):e1011651. doi: 10.1371/journal.pgen.1011651 (PMC11990764; doi:10.1371/journal.pgen.1011651)
Supplement: S2 Fig — Phased SNV + indel counts are plotted against parental age at conception. Linear regressions show stronger parental age effect than usually reported, likely due to small sample size. (PDF) [file pgen.1011651.s008.pdf]

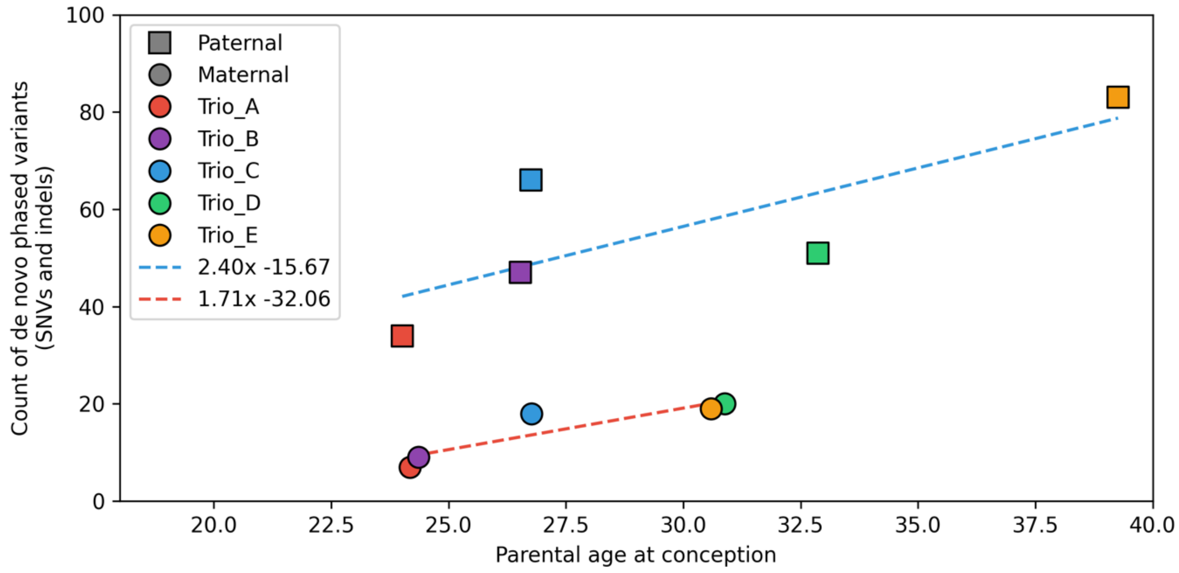

Supplementary Figure 1. Parental age effect on phased variants

Phased SNV + indel counts are plotted against parental age at conception. Linear regressions show stronger parental age effect than usually reported, likely due to small sample size.
