# Supplementary material for: Parental germline mosaicism in genome-wide phased de novo variants: Recurrence risk assessment and implications for precision genetic counselling
Source: PLoS Genet. 2025 Mar 31;21(3):e1011651. doi: 10.1371/journal.pgen.1011651 (PMC11990764; doi:10.1371/journal.pgen.1011651)
Supplement: S3 Fig — Signatures were extracted using the Signal interface (https://signal.mutationalsignatures.com/analyse2) based on signatures from Degasperi et al [50]. A. Proportion of trimer substitutions. B. Signatures extracted using the Signal interface. The SBS5 and SBS1 signatures are detected. TSB: transcriptional strand bias. The ‘TSB’ and ‘Deamination’ tags, as well as the ‘Age’ label, are standard annotations of the signatures and do not derive from the inputted DNMs. (PDF) [file pgen.1011651.s009.pdf]

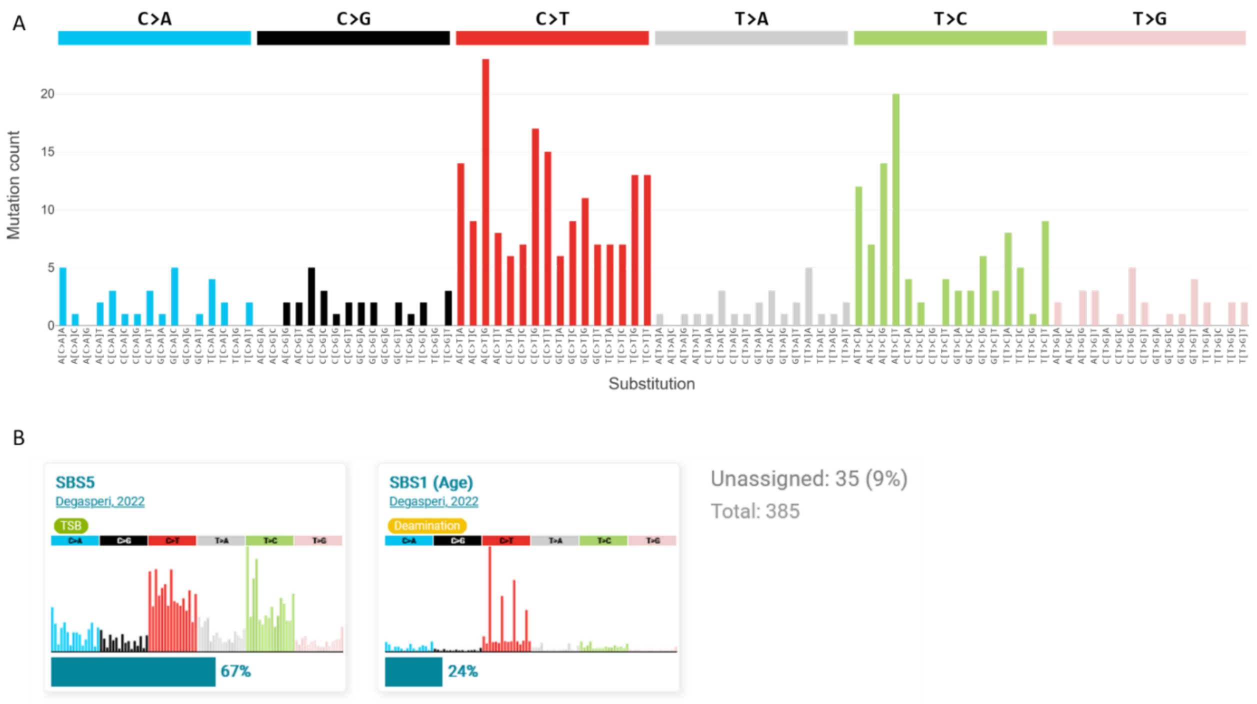


***Supplementary Figure 1. Single base substitution signatures extraction from the 385 de novo substitutions detected in this study***

Signatures were extracted using the Signal interface [1] (https://signal.mutationalsignatures.com/analyse2) based on signatures from Degasperi et al., ref[2].

A. Proportion of trimer substitutions

B. Signatures extracted using the Signal interface. The SBS5 and SBS1 signatures are detected. TSB: transcriptional strand bias. The 'TSB' and 'Deamination' tags, as well as the 'Age' label, are standard annotations of the signatures and do not derive from the inputted DNMs.

References:

1. Degasperi A, Amarante TD, Czarnecki J, Shooter S, Zou X, Glodzik D, et al. A practical framework and online tool for mutational signature analyses show inter-tissue variation and driver dependencies. Nat Cancer. 2020;1: 249–263. doi:10.1038/s43018-020-0027-5

2. Degasperi A, Zou X, Dias Amarante T, Martinez-Martinez A, Koh GCC, Dias JML, et al. Substitution mutational signatures in whole-genome–sequenced cancers in the UK population. Science. 2022;376: abl9283. doi:10.1126/science.abl9283
