# Supplementary material for: Parental germline mosaicism in genome-wide phased de novo variants: Recurrence risk assessment and implications for precision genetic counselling
Source: PLoS Genet. 2025 Mar 31;21(3):e1011651. doi: 10.1371/journal.pgen.1011651 (PMC11990764; doi:10.1371/journal.pgen.1011651)
Supplement: S4 Fig — Clusters were defined as variations separated by a maximum of 20kb and called by https://github.com/francois-lecoquierre/de_novo_tools/blob/main/DNM_cluster_by_sample.py. A. Genomic distribution of clusters. Regions enriched in maternal mutational clusters identified in the literature are shown in pink. Note that the only maternal cluster is present in one of these regions. It is also the largest cluster and contains the most variants (n=3, see C and D). Generated using Tagore software (https://github.com/jordanlab/tagore). B. Types of substitutions of clustered (n=13) versus non-clustered (n=372) variations. The drastic difference in Ti/Tv ratio between clustered and non-clustered variations recapitulates observations on larger trio studies. C. Characteristics of the 6 clusters detected. Note the higher prevalence of paternal clusters, in contrast to literature data in which the number of paternal clusters is equivalent to the number of maternal clusters. This difference is likely due to the small sample size. Of note, the phases of individual SNVs were concordant and have been merged in the “Parent Of Origin” column. D. Representation of the maternal cluster composed of 3 SNVs within the SMARCA2 gene in the hypermutable 9p region. UCSC euro session: https://genome-euro.ucsc.edu/s/francois.leco/RRMUT_maternal_cluster. (PDF) [file pgen.1011651.s010.pdf]

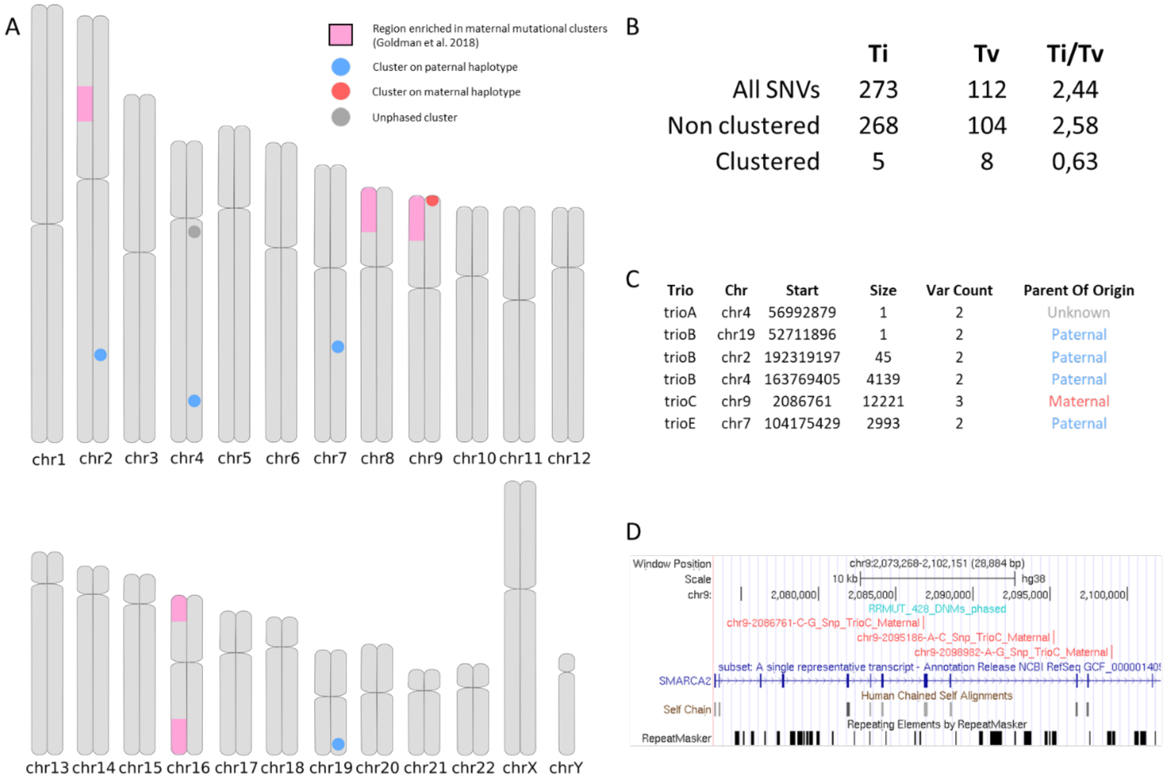

Supplementary Figure 1. Analysis of mutational clusters recapitulate known cluster properties

Clusters were defined as variations separated by a maximum of 20kb and called by https://github.com/francois-lecoquierre/de_novo_tools/blob/main/DNM_cluster_by_sample.py

A. Genomic distribution of clusters. Regions enriched in maternal mutational clusters identified in the literature are shown in pink (source: ref [1]). Note that the only maternal cluster is present in one of these regions. It is also the largest cluster and contains the most variants (n=3, see C and D). Generated using Tagore software (https://github.com/jordanlab/tagore).

B. Types of substitutions of clustered (n=13) versus non-clustered (n=372) variations. The drastic difference in Ti/Tv ratio between clustered and non-clustered variations recapitulates observations on larger trio studies [2].

C. Characteristics of the 6 clusters detected. Note the higher prevalence of paternal clusters, in contrast to literature data in which the number of paternal clusters is equivalent to the number of maternal clusters. This difference is likely due to the small sample size. Of note, the phases of individual SNVs were concordant and have been merged in the "Parent Of Origin" column.

D. Representation of the maternal cluster composed of 3 SNVs within the SMARCA2 gene in the hypermutable 9p region. UCSC euro session: https://genome-euro.ucsc.edu/s/francois.leco/RRMUT_maternal_cluster

References:

1. Goldmann JM, Seplyarskiy VB, Wong WSW, Vilboux T, Neerincx PB, Bodian DL, et al. Germline de novo mutation clusters arise during oocyte aging in genomic regions with high double-strand-break incidence. Nat Genet. 2018;50: 487–492. doi:10.1038/s41588-018-0071-6

2. Goldmann JM, Wong WSW, Pinelli M, Farrah T, Bodian D, Stittrich AB, et al. Parent-of-origin-specific signatures of de novo mutations. Nat Genet. 2016;48: 935–939. doi:10.1038/ng.3597
