# Supplementary material for: Parental germline mosaicism in genome-wide phased de novo variants: Recurrence risk assessment and implications for precision genetic counselling
Source: PLoS Genet. 2025 Mar 31;21(3):e1011651. doi: 10.1371/journal.pgen.1011651 (PMC11990764; doi:10.1371/journal.pgen.1011651)
Supplement: S5 Fig — (PDF) [file pgen.1011651.s011.pdf]

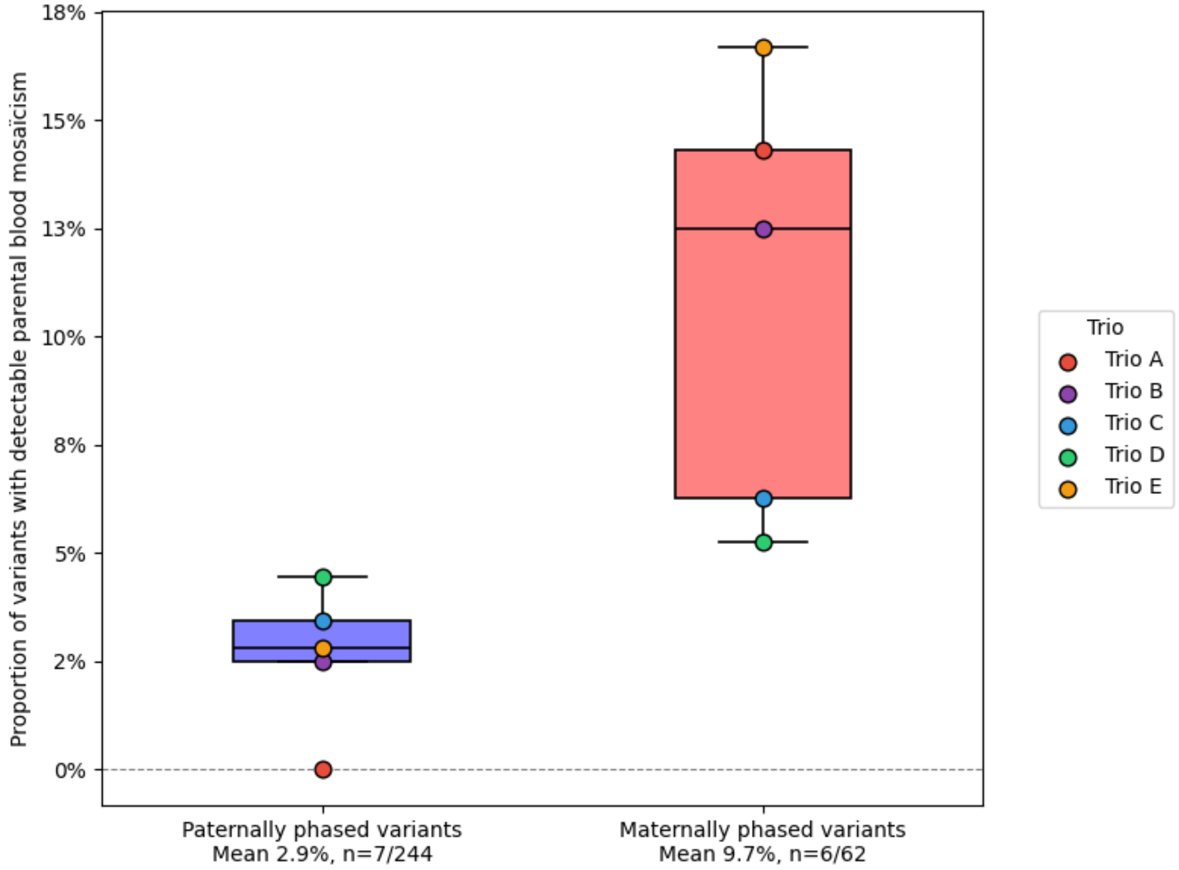

Supplementary Figure 1. Maternally phased variants display higher rate of parental blood mosaicism than paternally phased variants
