# Supplementary material for: Parental germline mosaicism in genome-wide phased de novo variants: Recurrence risk assessment and implications for precision genetic counselling
Source: PLoS Genet. 2025 Mar 31;21(3):e1011651. doi: 10.1371/journal.pgen.1011651 (PMC11990764; doi:10.1371/journal.pgen.1011651)
Supplement: S6 Fig — Two Allele VAF is defined by alt_read_count/(ref_read_count + alt_read_count) and does not integrates reads with other genotypes than ref and alt. (PDF) [file pgen.1011651.s012.pdf]

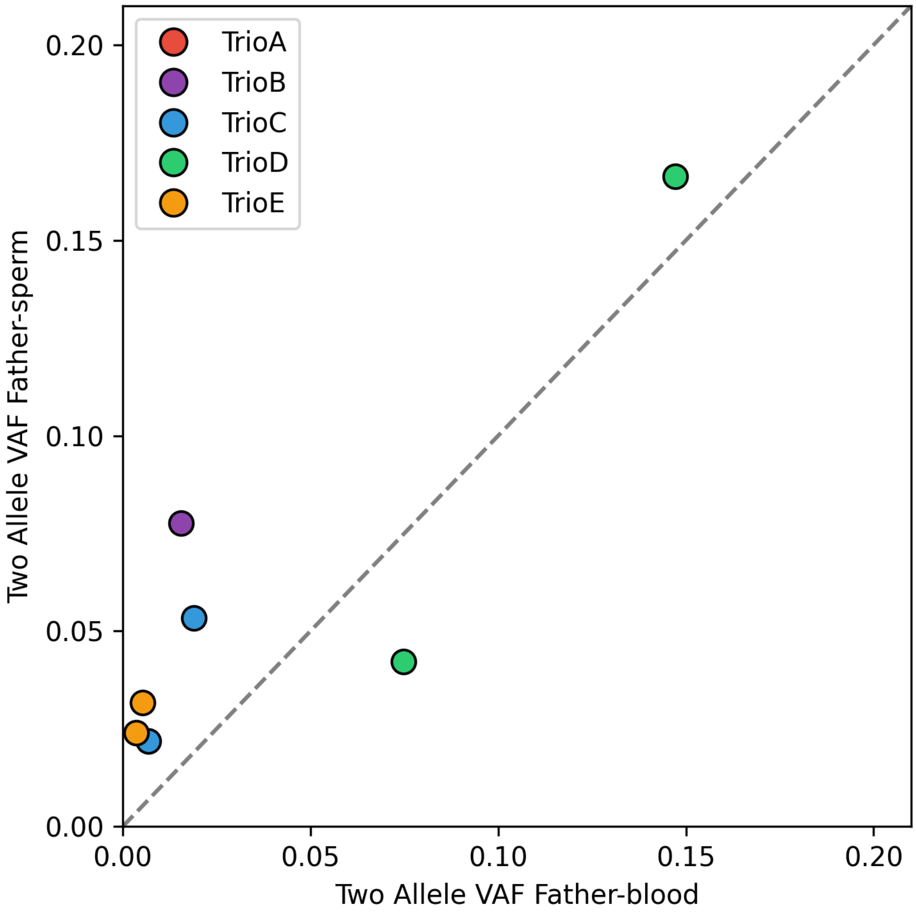

Supplementary Figure 1. Shared mosaicisms in fathers: correlation of VAF in sperm versus blood

Two Allele VAF is defined by alt_read_count/(ref_read_count + alt_read_count) and does not integrates reads with other genotypes than ref and alt.
