# Supplementary material for: Parental germline mosaicism in genome-wide phased de novo variants: Recurrence risk assessment and implications for precision genetic counselling
Source: PLoS Genet. 2025 Mar 31;21(3):e1011651. doi: 10.1371/journal.pgen.1011651 (PMC11990764; doi:10.1371/journal.pgen.1011651)
Supplement: S7 Fig — Early embryonic mosaicism was called for a subset of variants using two complementary VAF-related metrics within: (i) high depth smMIP data and (ii) phased long-read genome data. The variant chr2–43736835-C-A is displayed as an example for both these metrics in A) and B). A. smMIP pileup genotyping of the variant chr2–43736835-C-A showing a VAF that deviates from the 50% expected for a homogeneous genotype. B. The same variant as seen in the proband’s long read genome data. The de novo C>A transversion is phased on the maternal haplotype 2, corresponding to the purple reads. Ten maternally derived reads do not harbour the variant, highly suggesting mosaicism. From this example, the ratio of C>A-bearing purple reads over the total count of purple reads defines what we called the haplotype-specific VAF. This metrics is expected to be 100% in samples without post-zygotic mosaicism. C. Detection of child embryonic mosaicisms using the combination of the two VAF-related metrics. From all de novo variants, we extracted a subset of 163 variants with high quality genotypes in child’s sequencing data, both in Nanopore long-read genome data and high depth smMIP sequencing. More specifically, the filters included: (i) SNVs only, (ii) variants with a parental phase determined in long-read data, with a depth of at least 4x on the haplotype bearing the variant, and (iii) variants for which both the extension and ligation arms of the associated smMIP did not lie within a unique repeated element in “RepeatMasker” or “Human Self Chain Alignments” tracks from UCSC, since highly repeated elements were occasionally observed to slightly bias the smMIP-defined VAF by incomplete specificity. The smMIP VAF is centered on 0.5 for heterozygous de novo variants, as expected. The haplotype-specific VAF has been defined as the proportion of alt reads over (ref + alt) reads, only on the mutated haplotype defined by WhatsHap Haplotag. Since both smMIP and Nanopore sequencing exhibit noise in the [file pgen.1011651.s013.pdf]

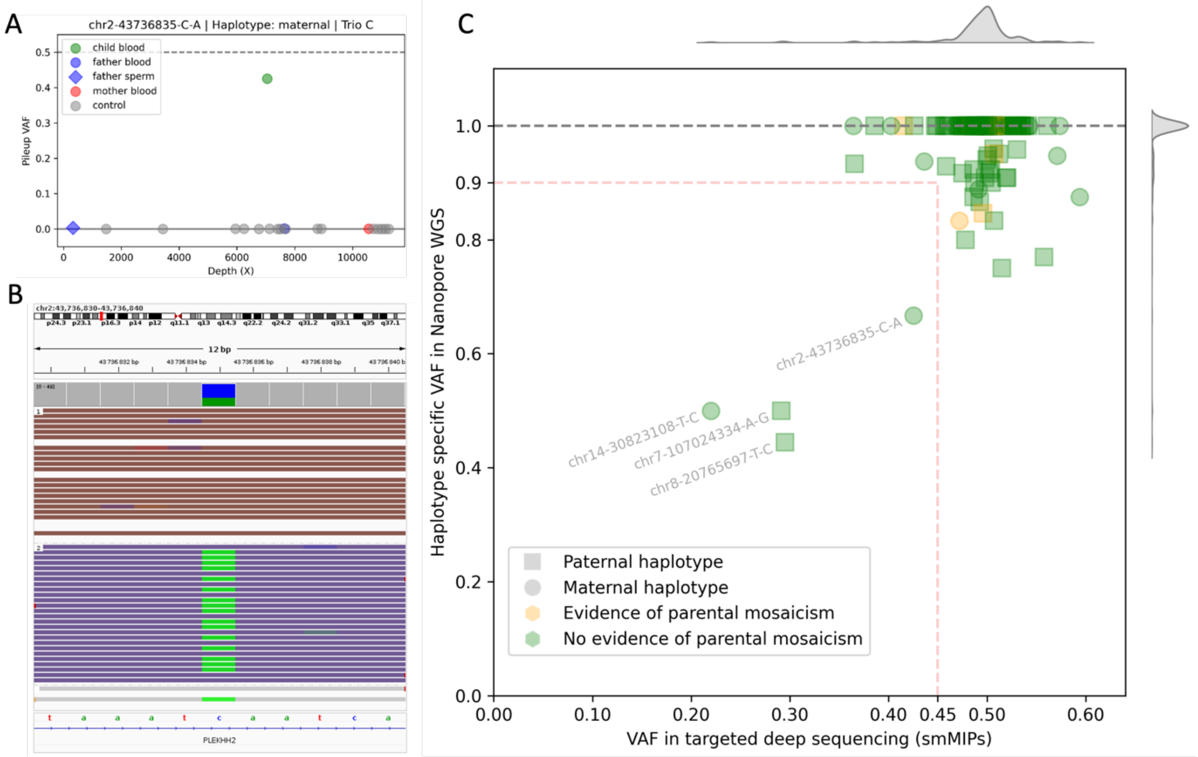

Supplementary Figure 1 : High evidence for four child embryonic mutations

Early embryonic mosaicism was called for a subset of variants using two complementary VAF-related metrics within: (i) high depth smMIP data and (ii) phased long-read genome data. The variant chr2-43736835-C-A is displayed as an example for both these metrics in A) and B).

A. smMIP pileup genotyping of the variant chr2-43736835-C-A showing a VAF that deviates from the 50% expected for a homogeneous genotype

B. The same variant as seen in the proband's long read genome data. The de novo C>A transversion is phased on the maternal haplotype 2, corresponding to the purple reads. Ten maternally derived reads do not harbour the variant, highly suggesting mosaicism. From this example, the ratio of C>A-bearing purple reads over the total count of purple reads defines what we called the haplotype-specific VAF. This metrics is expected to be 100% in samples without post-zygotic mosaicism.

C. Detection of child embryonic mosaicisms using the combination of the two VAF-related metrics. From all de novo variants, we extracted a subset of 163 variants with high quality genotypes in child's sequencing data, both in Nanopore long-read genome data and high depth smMIP sequencing. More specifically, the filters included: (i) SNVs only, (ii) variants with a parental phase determined in long-read data, with a depth of at least 4x on the haplotype bearing the variant, and (iii) variants for which both the extension and ligation arms of the associated smMIP did not lie within a unique repeated element in "RepeatMasker" or "Human Self Chain Alignments" tracks from UCSC, since highly repeated elements were occasionally observed to slightly bias the smMIP-defined VAF by incomplete specificity. The smMIP VAF is centered on 0.5 for heterozygous de novo variants, as expected. The haplotype-specific VAF has been defined as the proportion of alt reads over (ref + alt) reads, only on the mutated haplotype defined by WhatsHap Haplotag. Since both smMIP and Nanopore sequencing exhibit noise in the definition of the VAF, we considered high evidence mosaicisms as the variants with low VAF in both approaches. In this perspective, child mosaics were defined as variants with both a smMIP VAF < 0.45 and a haplotype-specific VAF < 0.9. These thresholds are indicated in pink dotted lines. Four variants meeting these criteria are highlighted. *De novo* variants with evidence for parental mosaicism are indicated in yellow and serve as negative controls since they are necessarily pre-zygotic.
