# Supplementary material for: Parental germline mosaicism in genome-wide phased de novo variants: Recurrence risk assessment and implications for precision genetic counselling
Source: PLoS Genet. 2025 Mar 31;21(3):e1011651. doi: 10.1371/journal.pgen.1011651 (PMC11990764; doi:10.1371/journal.pgen.1011651)
Supplement: S8 Fig — Samtools Mpileup was used to quantify the alt read count in parental WGS for the de novo variants included in this study and to compare it to the presence of a mosaicism as detected by deep sequencing. A. Counts of alt reads in parental genomes and mosaicism status. As expected, the presence of ≥ 2 reads in parental genomes appears highly predictive for parental embryonic mosaicism. These counts can be used to establish the performance of at least one alt read as an indicator of parental mosaicism. Recall is defined by the proportion of variants with mosaicism that have ≥ 1 alt read in parental WGS: 10/13 = 76.9%. Precision is defined by the proportion of variant with ≥ 1 alt read that are actually mosaic variants: 10/15 = 66.7%. B. Parental blood mosaicism: true blood VAF against alternate read count in 40x WGS. The 13 mosaicisms confirmed to be present in parental blood are plotted. Alternate read count from parental 40x WGS appears predictive of blood VAF detected by deep sequencing. (PDF) [file pgen.1011651.s014.pdf]

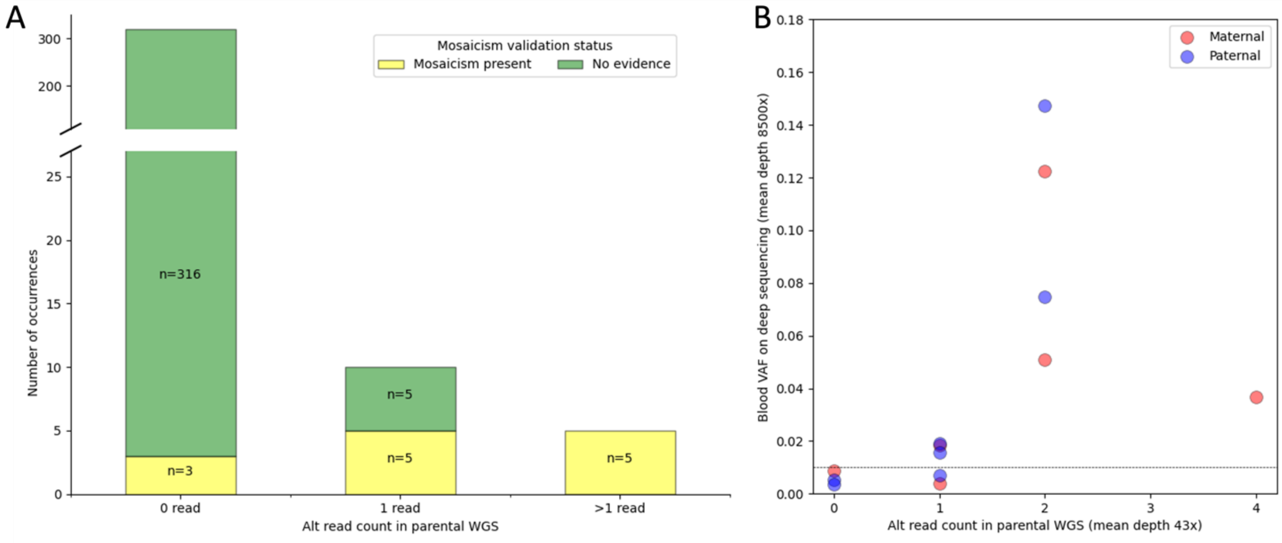

Supplementary Figure 1. Correlation between counts of alt reads in parental WGS and parental mosaicism status
Samtools Mpileup was used to quantify the alt read count in parental WGS for the de novo variants included in this study and to compare it to the presence of a mosaicism as detected by deep sequencing.

A. Counts of alt reads in parental genomes and mosaicism status. As expected, the presence of ≥ 2 reads in parental genomes appears highly predictive for parental embryonic mosaicism. These counts can be used to establish the performance of at least one alt read as an indicator of parental mosaicism. Recall is defined by the proportion of variants with mosaicism that have ≥ 1 alt read in parental WGS: 10/13 = 76.9%. Precision is defined by the proportion of variant with ≥ 1 alt read that are actually mosaic variants: 10/15 = 66.7%.
B. Parental blood mosaicism: true blood VAF against alternate read count in 40x WGS. The 13 mosaicisms confirmed to be present in parental blood are plotted. Alternate read count from parental 40x WGS appears predictive of blood VAF detected by deep sequencing.
