# Supplementary material for: Parental germline mosaicism in genome-wide phased de novo variants: Recurrence risk assessment and implications for precision genetic counselling
Source: PLoS Genet. 2025 Mar 31;21(3):e1011651. doi: 10.1371/journal.pgen.1011651 (PMC11990764; doi:10.1371/journal.pgen.1011651)
Supplement: S1 Text — (PDF) [file pgen.1011651.s015.pdf]

Prediction of recurrence risk using Decode Genetics' De Novo Mutation Recurrence Calculator

With the aim of comparing our detected recurrence risk for paternally phased variants to available predictors, we used Decode's De Novo Mutation Recurrence Calculator:

https://de-novo-risk.decode.is/.

This software, mentioned in Jónsson et al. [1], takes as an input a specific genomic variant and information regarding presence of variants in siblings, parent of origin, parental age at conception, sequencing depth and alt read count in parental genomes. It outputs a mean recurrence risk estimation. We modelled each paternally phased variant with the following parameters. We considered the mean paternal age of 30 years at conception in our five probands. Overall, 237 variants were identified with 0 alt reads in a mean of 41x fathers, 5 variants were identified with 1 alt read in a mean of 41x, and 2 reads were identified with 2 alt reads in 38x. These three situations translated to 0.43%, 2.68% and 9.27% recurrence risk, which averaged at 0.55%.

Reference:

1. Jónsson H, Sulem P, Arnadottir GA, Pálsson G, Eggertsson HP, Kristmundsdottir S, et al. Multiple transmissions of de novo mutations in families. Nat Genet. 2018;50: 1674–1680. doi:10.1038/s41588-018-0259-9
