# Supplementary material for: Parental germline mosaicism in genome-wide phased de novo variants: Recurrence risk assessment and implications for precision genetic counselling
Source: PLoS Genet. 2025 Mar 31;21(3):e1011651. doi: 10.1371/journal.pgen.1011651 (PMC11990764; doi:10.1371/journal.pgen.1011651)
Supplement: S2 Text — (PDF) [file pgen.1011651.s016.pdf]

How to assess the risk of recurrence for maternally derived variants?

We define the recurrence risks of paternally and maternally phased variants as follows:

$$RR_{pat} = \frac{N_{\text{at risk DNM from father}}}{N_{\text{total paternal DNMs}}} \times \overline{\text{VAF}}_{\text{sperm}} \qquad RR_{mat} = \frac{N_{\text{at risk DNM from mother}}}{N_{\text{total maternal DNMs}}} \times \overline{\text{VAF}}_{\text{oocytes}}$$

Where $N_{\text{at risk DNM from father}}$ and $N_{\text{at risk DNM from mother}}$ represent the count of germinal mosaic variants from each parent and $\overline{\text{VAF}}_{\text{sperm}}$ and $\overline{\text{VAF}}_{\text{oocytes}}$ their associated average VAF in sperm and oocytes respectively. For the paternal branch, all parameters can be observed and retrieved from our data. Since maternal and paternal mosaic events are intended to occur independently from sex differentiation, it is likely that the absolute count of oocyte mosaics are similar to sperm mosaics, with similar VAFs. Therefore, we can assume that $N_{\text{at risk DNM from mother}}$ and μ_{oocytes} can be retrieved from the paternal branch, namely:

$$RR_{pat} = \frac{N_{\text{at risk DNM from father}}}{N_{\text{total paternal DNMs}}} \times \overline{\text{VAF}}_{\text{sperm}} \qquad RR_{mat} = \frac{N_{\text{at risk DNM from father}}}{N_{\text{total maternal DNMs}}} \times \overline{\text{VAF}}_{\text{sperm}}$$

Eventually, only the paternal and maternal denominators vary between both equations, which leads to:

$$RR_{mat} = RR_{pat} \times \frac{N_{\text{total paternal DNMs}}}{N_{\text{total maternal DNMs}}}$$
